# Kinetics of severe acute respiratory syndrome coronavirus 2 infection antibody responses

**DOI:** 10.3389/fimmu.2022.864278

**Published:** 2022-08-05

**Authors:** Yajie Lin, Jiajie Zhu, Zongming Liu, Chaonan Li, Yikai Guo, Ying Wang, Keda Chen

**Affiliations:** ^1^ Shulan International Medical College, Zhejiang Shuren University, Hangzhou, China; ^2^ Key Laboratory of Oral Biomedical Research of Zhejiang Province, Zhejiang Provincial Clinical Research Centre for Oral Diseases, Cancer Centre of Zhejiang University, Stomatology Hospital, School of Stomatology, Zhejiang University School of Medicine, Hangzhou, China

**Keywords:** SARS-CoV-2 infection, antibody responses, kinetics, VOC strains, review

## Abstract

Severe acute respiratory syndrome coronavirus 2 (SARS-CoV-2) has spread rapidly throughout the world, causing severe morbidity and mortality. Since the first reports of Coronavirus disease 2019 (COVID-19) in late 2019, research on the characteristics of specific humoral immunity against SARS-CoV-2 in patients with COVID-19 has made great progress. However, our knowledge of persistent humoral immunity to SARS-CoV-2 infection is limited. The existence of protective immunity after infection will affect future transmission and disease severity. Therefore, it is important to gather knowledge about the kinetics of antibody responses. In this review, we summarize the information obtained so far on the characteristics and kinetics of the SARS-CoV-2 infection of specific humoral immune response, especially in neutralizing antibodies and their relationship with disease severity. In addition, with the emergence of variants of concern, we summarize the neutralizing effect of specific humoral immunity on variants of concern after the initial SARS-CoV-2 infection and vaccination.

## Introduction

**Figure 1:**
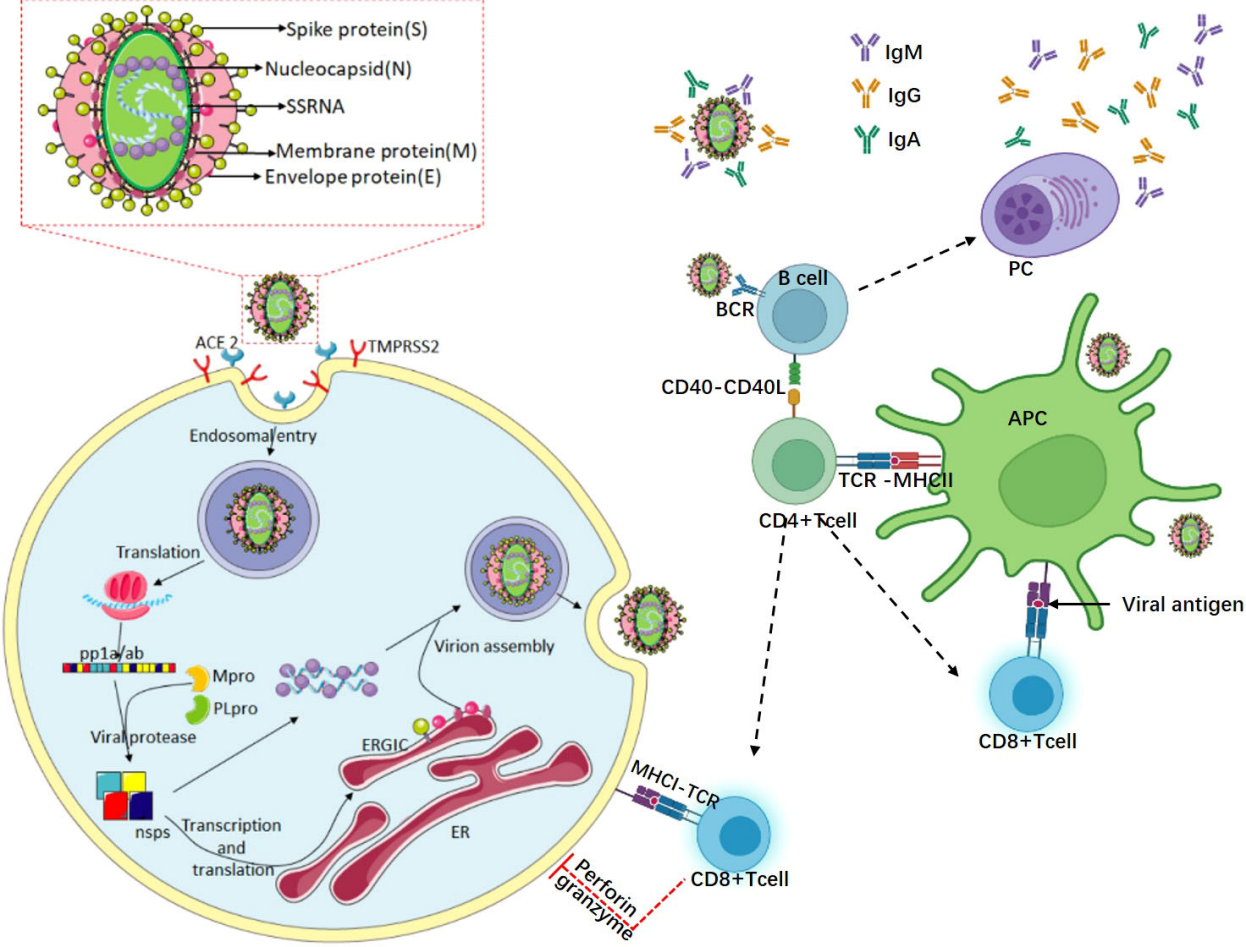
The life cycle of SARS-CoV-2 and the specific immune response to the virus. The virus particles of SARS-CoV-2 are composed of four major proteins, envelope (E), spike glycoprotein (S), membrane (M), and the nucleocapsid (N). SARS-CoV-2 promotes virus invasion through the interaction between the S protein, hACE2, and TMPRSS2. After successful invasion, the viral genome RNA is released in the cytoplasm, and then initiates the translation of the genome. The translated polymeric proteins are cleaved by Mpro and PLpro to generate non-structural proteins (nsps). These nsps are involved in viral replication and transcription. With the nucleocapsid (N) protein-encapsidated genomic RNA, the structural proteins are translocated to the endoplasmic reticulum (ER) membrane and assembled into a new virus in the ER-to-Golgi intermediate compartment (ERGIC). Finally, virus particles are secreted from infected cells *via* exocytosis. APC, B cells, CD4+ T cells, and CD8+ T cells are the main cells that participate in adaptive immunity. The virus is recognized by professional antigen-presenting cells (such as dendritic cells and macrophages), and the viral peptides are presented to CD4+ T cells through the major histocompatibility complex (MHCII) class II. Then, the CD4+ T cells differentiate into a series of helper cells, which in turn help activate CD8+ T cells and B cells. CD8+ T cells can kill infected cells with the assistance of CD4+ T cells and MHCI. Simultaneously, B cells transform into plasma cells to produce neutralizing antibodies to prevent the virus from invading again.

Coronavirus disease 2019 (COVID-19) is an infectious disease caused by severe acute respiratory syndrome coronavirus 2 (SARS-CoV-2). The first case was detected in Wuhan, Hubei, China at the end of 2019, and then it spread rapidly, forming a large-scale global outbreak. SARS-CoV-2 is an enveloped single-stranded, positive-sense RNA virus, belonging to the Betacoronavirus genus (1). There are 14 open reading frames (ORFs) in the SARS-CoV-2 genome, encoding four structural proteins: the spike glycoprotein (S), envelope (E), membrane (M), and nucleocapsid (N); 16 nonstructural proteins (NSP 1–16), and nine accessory proteins (ORF3a, ORF3b, ORF6, ORF7a, ORF7b, ORF8, ORF9a, ORF9b, ORF10) (2, 3). The viral capsid formed by the N protein wraps the viral genome, while the E and M proteins participate in the assembly and release of the virion. The S protein is the key protein for viral invasion of cells (4). The S protein consists of S1 and S2 subunits. S1 folds into the N-terminal domain (NTD), receptor-binding domain (RBD), and two C-terminal domains (CTDs) (5, 6). The RBD of S1 interacts with human angiotensin-converting enzyme (hACE2) to promote fusion between the cell membrane and the virus envelope, and the virion can reproduce in the cells (3, 5–10). **Figure 1** shows a schematic diagram of the viral infection and replication.

The immune system can be broadly divided into the innate immune system and the adaptive immune system. Innate immunity is the first line of defense of the immune system (11). The innate immunity system restricts virus replication in infected cells and produces an antiviral state in the local tissue environment, which slows down the replication and spread of the virus. In addition, the innate immune response is critical to trigger the adaptive immune response (12). Adaptive immunity takes time to generate enough virus-specific cells to control the infection. Adaptive immunity involves three main cell types: CD8+ T cells, CD4+ T cells, and B cells (12). After the virus enters the tissue cells, viral peptides are presented to CD8+ cytotoxic T cells through the class I major histocompatibility complex (MHC) protein, and CD8+ cytotoxic T cells can produce cytotoxic effects on virus-infected tissue cells and induce apoptosis through perforin, granzyme, and other mechanisms (13). Professional antigen-presenting cells (e.g., macrophages and dendritic cells) recognize viruses and viral particles, and the class II major histocompatibility complex (MHCII) presents viral peptides to CD4+ T cells. In patients with SARS-CoV-2 infection, CD4+ T cells usually differentiate into T helper type 1 (Th1) cells and T follicular helper (Tfh) cells, which have the ability to instruct B cells, help CD8+ T cells, and recruit innate immunity cells. Recognition of the virus by B cells leads to their activation and interaction with CD4+ T cells (13). After activation, naive B cells proliferate and differentiate into plasma cells, secreting antibodies to prevent the entry of viruses outside the cells (**Figure 1**). The seroconversion time of antibodies varies according to the difference of the target antigen and the subtype of the antibody (12). In the fight against the virus, anti-SARS-CoV-2 antibodies are essential. Proper neutralization will greatly reduce the number of viruses that infect ACE2 receptor-expressing cells. Meanwhile, the presence of antibodies also provides immunity against reinfection.

## Dynamic changes in antibodies

In the process of B cell differentiation, a unique variable chain is produced through a series of complex gene rearrangements and can be subjected to somatic hypermutation after exposure to the antigen to allow affinity maturation (14). Different variable domains can specifically recognize different parts of the virus. The nucleocapsid protein, spike glycoprotein, and S protein fragments (S1, RBD, S2) of SARS-CoV-2 are the main antigens that induce antibody responses (15). The Spike protein is the main target of SARS-CoV-2 neutralizing antibodies, and the Spike RBD is the target of > 90% of neutralizing antibodies (12). The constant domain includes a fragment crystallization (Fc) portion that mediates the biological effect of an antibody by binding to cell surface receptors (Fc receptors) on circulating leukocytes, macrophages, and natural killer cells (16). Human antibodies are divided into five isotypes according to their constant domain: Immunoglobulin (Ig)M, IgG, IgA, IgD, and IgE. Different isotypes play a diverse roles in the process of anti-viral infection according to the characteristics of their structure, generation time, distribution, and half-life (6).

**Figure 2:**
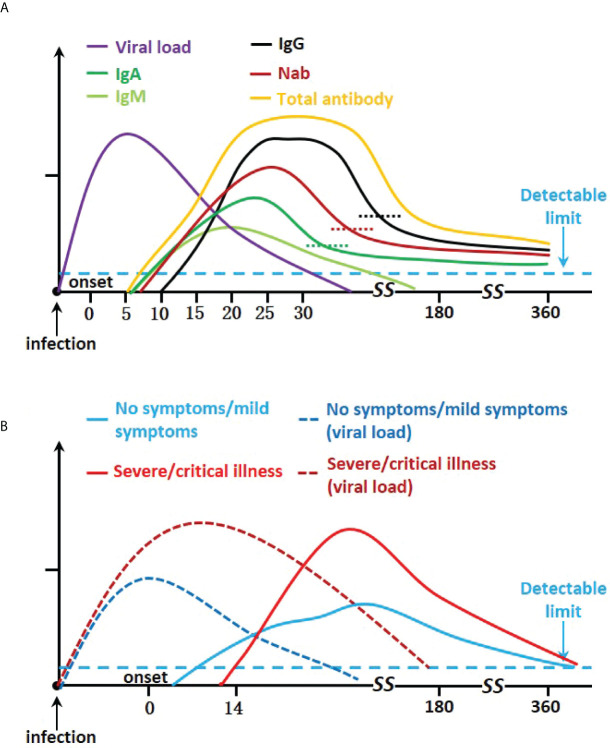
Kinetics severe acute respiratory syndrome coronavirus 2 infection antibody responses. **(A)** Graph showing IgA, IgM, IgG, Nab, viral load, and total neutralizing antibody trends. **(B)** Graph of disease severity, viral load, and total neutralizing antibodies.

Almost all SARS-CoV-2 transmission models assume that the immunity generated by the infection has a protective effect against reinfection for a duration of at least 1 year (17). To form a lasting and effective immunity to the virus, the dynamics of antibody changes are very important. The study of the dynamic changes of antibody levels mainly involves the seroconversion time of different isotypes, the change of their concentrations with time, and how long they last. The study of antibody duration closely follows the time course of the first known infected patients. In this section, we summarize relevant information on the dynamic of antibodies (**Figure 2A** and **Supplementary Table 1**).

### IgM

IgM is the first immunoglobulin expressed during B cell development. Monomeric IgM is expressed on the surface of naive B cells. After initiating adaptive immune defense against pathogens, plasma cells first secrete multimeric (usually pentameric) IgM into the blood. IgM works by opsonizing (coating) antigens to destroy and fix complement (16). Arkhipova-Jenkins et al. comprehensively analyzed a number of studies on post–SARS-CoV-2 infection antibodies and showed that the mean detection time of IgM was about 7 days after disease onset, the peak was about 20 days into the disease, and it decreased at about 27 days (moderate-strength evidence) (18). There are relatively few studies tracking the duration of IgM, possibly because IgM mostly works in the early stage and has a short half-life. Wheatley et al. found that S-IgM decays faster in the early stage, with a half-life of 55 days, and slower in the later stage of recovery, with a half-life of 118 days (19). The RBD-IgM level dropped by about 6 folds within 3 months after discharge and then turned negative (20). The study of patients from Wuhan found that S and N-IgM peaked within 1–2 months after the onset of symptoms, then rapidly decreased, and was less than the cut-off value at 5–6 months. At 1 year, the residual positive rates were 5.3% and 1.3%, respectively (21). Our previous research found similar results. At 1 year (213–416 days post-symptom onset, PSO), the residual positive rate of RBD-IgM was 7.1%, whereas S2 and N-IgM were both 0% (22).

### IgA

IgA is mainly secreted in the form of dimeric molecules, which called secreted IgA (sIgA), and a small amount of IgA is also secreted into the serum as a monomer. The mucosal surface is the first entry point of SARS-CoV-2. sIgA is mainly found in the mucosa, tears, and saliva and plays a vital role in the immune defense of the mucosal surface (16). sIgA neutralizes invading viruses by binding to the Spike protein on the surface of SARS-Cov-2, thereby, preventing the virus from colonizing the genitourinary tract, gastrointestinal tract, and respiratory tract (23). The IgA response that appears in the early stage is stronger than the IgM response and lasts longer. S-IgA could be detected as early as 5–8 days PSO (5, 24). The median conversion time of IgA, targeting S1 or S, was 11–13 days PSO (24, 25). The specific IgA, targeting different antigens (S, RBD, N), peaked at about 1 month (16–30 days PSO) and then declined (26, 27). Cross-sectional analysis showed that the S-IgA response could be described using a short one-phase decay model incorporating a prolonged plateau phase, with initial half-lives of S-IgA and RBD-IgA of 14 days and 27 days, respectively, after which their levels declined relatively gently. In longitudinal analysis, the half-life of S-IgA was about 210 days and that of RBD-IgA was 74 days (28). Wheatley et al. found that S-IgA showed a similar two-phase decay, with a half-life of 42 days in the early stage and a slower decay in the later stage of recovery (19). A 1-year follow-up analysis found that the RBD-IgA declined by about 3 folds over the first 3 months, remained steady up to 6 months, and became negative at 12 months (20). Another study showed that the S1-IgA positive rate of patients with mild COVID-19 was 92% at 6 weeks after the onset, which dropped to 78% after 6 months with a decrease in titer of about 50%, and while at 12 months, there was still a 78% positive rate and the titer was not significantly different from that at 6 months (29). Sterlin et al. tested the antibody titers in the serum, saliva, and bronchoalveolar lavage fluid of patients with COVID-19 and found that IgA in saliva lasted longer than that in serum, and the concentration of IgA in saliva was higher than that of IgG. IgA decreased significantly in the serum at 1-month PSO, while neutralizing, IgA could still be detected in the saliva 49–73 days PSO, but not at a later time point (189–230 days PSO) (30). Our previous studies have shown that N-IgA rises fastest in the early stages of infection, with a 30.4% seroprevalence rate at 1-week PSO. During the follow-up period (213–416 days POS), the positive rate of IgA antibodies (targeting RBD, S2, and N) dropped below 10% (22).

### IgG

IgG is the most common immunoglobulin in the blood that usually works in the later stages of the humoral immune response. Its small size (monomeric), high diffusivity, and high affinity, make IgG the main type of antibody involved in neutralization, opsonization, and activation of the complement cascade (5, 16). In addition, because IgG has a durable half-life and is associated with memory B cells, it plays a role in long-lasting immunity (6). The seroconversion time of IgG is later than that of IgM. Based on a number of studies, it is suggested that IgG can be detected at about 12 days PSO, reaches a peak at about 25 days, and then remains relatively stable (moderate-strength evidence) (18).

The IgG targeting the nucleocapsid and the IgG only recognizing RBD have a shorter duration than the IgG targeting the intact Spike protein (28). Dan et al. conducted a cross-sectional study on 188 patients with COVID-19 (detection time 6–240 days PSO). A total of 254 samples were analyzed, among which 43 samples were tested at more than or equal to 6 months after infection. The best-fit curve indicated that continuous decay was the preferred model, which speculated that the half-lives of S-IgG, RBD-IgG, and N-IgG were about 140 days, 83 days, and 68 days, respectively. Longitudinal studies using the subset of subjects donated at two or more time points showed similar trends, the half-lives of IgG targeting S, RBD, and N were 103 days, 69 days, and 68 days, respectively (28). Terpos et al. collected the anti-SARS-CoV-2 antibody data of patients with COVID-19 longitudinally at three time points (median 2.1, 5.6, and 8.4 months), and found that antibodies targeting four antigens (the trimeric Spike, Spike-RBD, Nucleocapsid, and the Nucleocapsid RNA Binding Domain) decreased within 8 months after infection, and showed a two-phase decay with a more obvious decrease during the first 6 months PSO. It was estimated that the half-lives of IgGs against Spike, Spike-RBD, nucleocapsid, and Nucleocapsid RNA Binding Domain were 97, 62, 47, and 47 days, respectively, and the estimated half-life was significantly longer after 6 months (31). The Co-Stars study recruited 3,679 healthcare workers prospectively and performed monthly serological testing for a maximum of 7 months. Under the most pessimistic assumptions of continuous exponential decay, the half-lives for IgG targeting S, RBD, and N were predicted to be 126, 102, and 60 days. In addition, at 200 days PSO, 75% of individuals had detectable N antibodies, > 95% of individuals had detectable S antibodies, and it was predicted that S antibodies would remain detectable in 95% of individuals until 465 days (32). Wheatley et al. distinguished S-specific antigens in more detail and reported that the half-life of IgG targeting S-specific was 229 days, RBD was 126 days, S1 was 115 days, and S2 was 344 days, whereas N-specific IgG decayed significantly faster, with a half-life of 71 days (19).

A long-term follow-up studies showed that the IgG overall seropositivity rate of convalescent individuals remained relatively stable. A 1-year longitudinal study of patients from Wuhan found that RBD-IgG titers decreased over time, whereas, after 9 months, the geometric mean titer (GMT) began to stabilize at 64.3% of the initial level. The GMT of RBD-IgG at the 12th month after diagnosis decreased by 69.9% compared with that in the first month, and the positive rate exceeded 70%. Patients with a higher initial titer decayed faster; however, the RBD-IgG titer was still higher than that of patients with a lower initial titer (15). Other longitudinal studies came to similar conclusions, that RBD-IgG declined rapidly by about 3 folds within the first 3 months, further declined by about 5 folds at 6 months, and remained stable from 6 months to 1 year (20, 33). As for S1-IgG, the S1-IgG enzyme-linked immunosorbent assay (ELISA) test of patients with apparent pneumonia remained positive 1 year after infection, and some patients with subtle pneumonia (33.3%) and asymptomatic patients (28.6%) had a negative result (34). Choe et al. reported that the detectable rate of S1-IgG in patients with mild COVID-19 after 1 year was 57.7% (35). Longitudinal studies found that the S1-IgG positive rate of patients with mild COVID-19 dropped from 95% at 6 weeks to 70% at 6 months after the onset, with the titer decreasing by about 50%, while at 12 months, 66% of patients were still positive, and the titer remained stable (29). Guzmán-Martínez et al. predicted that the maximum duration of S1-IgG in SARS-CoV-2 infected patients could reach 744 days based on linear mixed models (36). Similar to RBD-IgG, S-IgG declined rapidly within the first 6 months and remained stable until 12 months (20), whereas the N-IgG declined significantly during this period (33, 37), with only 20% remaining seropositive after 1 year (37). Consistent with this research, Haveri et al. reported that S-IgG persisted in 97% of subjects for at least 13 months after infection, but only 36% had N-IgG. Studies also reported a high seroprevalence rate of both S-IgG and N-IgG after 1 year. The S-IgG and N-IgG seroprevalence rates of convalescent individuals were 90.8% and 88.2%, respectively (21), and in patients with mild COVID-19, 84.6% and 82.7% of individuals still had detectable S-IgG and N-IgG, respectively (35). At 14 months, the anti-spike-receptor binding domain (S-RBD) IgG persisted in 96.8% of mildly and moderately infected subjects (38). S2 was not routinely evaluated in most serological tests, and our study found that S2 IgG maintained a high positive rate during follow-up. The seropositivity rate of S2-IgG (85.7%) at 1 year (213–416 days POS) was higher than that of RBD-IgG and N-IgG at 19.0% and 52.4%, respectively (22).

### Neutralizing antibodies

Not all antibodies that bind to pathogenic particles have a neutralizing effect. Neutralizing antibodies (Nabs) that can prevent viruses from infecting cells by affecting virus surface molecules have vital functions in restricting virus replication in host cells and reduce virus infectivity (39). The specific binding of non-neutralizing antibodies might promote SARS-CoV-2 to enter cells through Fc-Fc receptor interaction and cause antibody-dependent enhancement (ADE) (23).

Research has shown that the dynamic changes of Nabs are quite different. Nabs can be detected 6–15 days after disease onset (40), peak at 14–45 days (26), and then decrease (18, 41). Nabs showed a two-phase decay, rapidly decreasing within 1–2 months, which was related to the rapid decline of IgM and IgA, and then a slow decrease, which was consistent with the slow decline of IgG (39). In a cross-sectional study, the half-life of the one-phase decay of Nabs was 27 days, the half-life of continuous decay was estimated to be 114 days, and the half-life in the longitudinal analysis was 90 days (28). The longitudinal study by Wheatley et al. also found that the decline of neutralization titer conforms to a two-phase decay model (23). The half-life of up to day 70 PSO was 55 days, and then the decay slowed down, with an estimated half-life of 519 days. This was consistent with the two-phase decay of immune plasma’s capacity to inhibit the interaction of viral RBD with hACE2 receptors (19). Another study proposed a differential half-life (35). The half-life of Nabs in the first 6 months was 47 days, and the half-life from 6 to 8 months was reduced to 27 days. The study had relatively few follow-up data at 8 months; therefore, this estimation should be deemed explorative. In addition, this difference might also be related to the differences in the calculation methods between studies (31).

The titer of Nabs decreases over time and their protective ability also declines; however, the protective effect of Nabs against serious diseases might last longer. A study of the earliest patients in Wuhan found that the neutralizing activity was only detected in about 43% of patients at 1-year PSO, and most individuals had low Nab titers (21). Other studies have shown that 48%–57.7% of patients with mild COVID-19 had positive neutralizing activity 1 year after infection (29, 35). Haveri showed that in a population with a lower proportion of elderly people, Nabs against wild-type (WT) virus persisted in 89% of patients for at least 13 months after infection (42). Our research revealed a stable positive rate of Nabs among the samples taken from patients with the longest follow-up (213–416 days POS), among which 95.2% remained positive. This might be related to a large number of severe and critically ill patients included in the study (44.1% and 29.4%, respectively) (22). Differences in the included population would affect the results of the duration of Nabs.

The detectable Nabs might not be sufficient to provide protection, and the Nab titer is of more concern. The detection methods of the Nab titer in different studies are quite heterogeneous; therefore, it is difficult to compare the absolute magnitude of the responses. The Nab titer decreased significantly in the first 6 months after infection, and the decline was slower between 6 months and 1 year (20, 34), with no significant difference being detected between Nab titers at 12 months and 6 months after the initial infection in patients (20, 33). A study on healthcare workers shown that the Nab titer at 1 year was not significantly different from the titers at 1–3 months after the initial infection, and Nabs provided protection against re-infection 1 year after the first infection for 56.1% of healthcare workers who had not been vaccinated (43).

In addition, a decline in antibody titers might not indicate a decrease in protection. A mature SARS-CoV-2 antibody response enhances cross-neutralization toward circulating variants. Moriyama. et al. indicated that although the antibody titer and total neutralizing activity declined, neutralization potency (neutralizing ability per virus-binding antibodies) and neutralization breath (cross-neutralizing ability to variants per neutralizing antibodies) increased overtime after 3 months PSO (44). Antibodies induced by SARS-CoV-2 accumulated somatic mutations in the germinal center, which could increase the affinity for cognate antigens. Compared with the antibodies isolated at 1.3 months, those isolated at 6.2 months had higher potency (45). The persistent viral antigen deposition during the convalescence period mediated the progressive supply of high-affinity antibodies, while low-affinity antibodies decayed over time. The increase in neutralization breath might be related to the durable IgG response that was resistant to RBD mutations (44). Khoury et al. estimated that about 20% of the average convalescent neutralizing antibody titer could provide 50% protection from symptomatic COVID-19, and a lower Nab titer was required to provide 50% protection from severe COVID-19, at approximately 3% of the average convalescent Nab titer (46). According to the threshold, 17% of COVID-19 survivors obtained 50% protection against detectable WT SARS-CoV-2 re-infection and 87% of participants received 50% protection against severe disease of up to 1-year post infection (47). Lau et al. estimated that in symptomatic patients, the levels of Nabs conferring 50% protection would be maintained for around 990 days PSO (48).

## The relationship between antibody kinetics and disease severity

There is a wide heterogeneity in the severity of COVID-19 disease, and the patient’s antibody response varies. Available studies suggest that irrespective of disease severity, the majority of patients mount a robust adaptive immune response. Higher levels of antibodies and delayed humoral response are associated with severity (49–51) (**Figure 2B**).

In general, compared with patients with asymptomatic or mild infections, the levels of several antibody isotypes and Nabs in patients with severe and critical SARS-CoV-2 are higher. In the acute phase, the IgG seroprevalence was not significantly different between asymptomatic patients and symptomatic patients at 81.1% and 83.8%, respectively. However, the IgG level of asymptomatic patients was significantly lower relative to the symptomatic group, and more asymptomatic patients became seronegative in the early convalescent phase (52). Rijkers et al. compared patients with severe and mild disease and showed that only 87% of patients with mild disease had detectable antibodies (IgA and IgG) at 21–28 days PSO, with a significantly lower titer than that of patients with severe disease (53). Yu et al. reported similar results, compared with patients with non-severe disease, those with severe disease had significantly higher levels of IgA and IgG; however, there was no statistically significant difference in IgM levels (25). Meanwhile, Lynch et al. reported that compared to those with milder disease, patients admitted to the ICU had enhanced peak IgM levels at 6–20 days and higher peak IgG levels after 5 days (54). In addition, the antibody response of patients with severe COVID-19 was not only higher but also had broader SARS-CoV-2 polyantigenicity, whereas patients with only spike reactivity tended to exhibit mild or moderate symptoms (55).

In patients with mild or asymptomatic disease, the neutralizing activity decreased insignificantly within 6 months after diagnosis or symptoms onset. In comparison, the titers of Nabs in hospitalized patients were higher and followed a two-phase decay pattern, with a rapid decline at first and a slowdown after day 80. Nonetheless, hospitalized patients still had higher neutralizing activity than patients with mild disease at 6 months (50). However, Xiang et al. found that 1 year after the onset of symptoms, in convalescent patients, the proportion of neutralizing antibodies in patients who had non-severe disease was higher than that in severely ill patients at 44% and 39.1%, respectively (21). Feng et al. showed Nab levels were only transiently higher as a result of severe symptoms during the earlier convalescence stage, which then decreased from 3 months to 1 year (20).

Another view is that the clinical trajectory and results are not related to the cross-sectional level of the antibody, but related to the timing of antibody production and antibody kinetics. Ren et al. found that the role of antibody titers in predicting the severity of COVID-19 was limited; however, a delayed antibody response was a risk factor for disease severity (56). A study reported that IgG and IgM levels in critical cases were lower than those in severe and moderate cases at the initial stage of onset. Compared with the moderate group, the increase in IgM in the severe group was delayed, whereas in the critical group, the production of both IgM and IgG antibodies was delayed (57). Lucas et al. found that high S-IgG was associated with increased disease severity; however, the overall humoral response of patients who died was not higher than that of live discharged patients. Patients who died showed a delayed humoral response, and the delayed seroconversion kinetics was associated with impaired virus clearance. In addition, early Nab production (< 14 days PSO) was related to the improvement of clinical symptoms and the reduction of mortality, and compared with the late Nab production group, the maximum viral load over the entire course was lower (58). Zhou et al. also showed that compared with discharged patients, the anti-S, anti-RBD, and anti-neutralizing antibodies levels in patients who died were significantly lower in the first week PSO, increased in week 2, and then tended to be consistent with those of the discharged patients (59).

There are many factors that affect the severity of COVID-19 infection and neutralizing antibody levels, such as age, gender, comorbidities, etc. The older the age, the higher the risk of the fatal disease. A positive correlation was found between age and antibody levels (60). This may be due to the decreasing abundance of naive T cells and professional antigen-presenting cells in the elderly, delaying adaptive immune responses, and resulting in a high-viral burden that derives higher antibody titer (12, 61). Male and female patients have different immune capabilities at the early stage of SARS-CoV-2 infection. During SARS-CoV-2 infection, T cell activation was stronger in female patients than in male patients (12, 62, 63). By contrast, male patients are at higher risk of severe COVID-19 and have higher levels of neutralizing antibodies compared with female patients (60, 63). Additionally, patients with comorbidities (such as cardiovascular disease, obesity, and type 2 diabetes mellitus) have an increased rate of severe and fatal COVID-19 disease (62). At 6 months PSO, patients with comorbidities have higher levels of S-IgG and neutralizing antibody (64), which may be related to immunosenescence and a dysfunctional immune system.

## Neutralization of virus variants

Since onset of the new coronavirus epidemic, a variety of SARS-CoV-2 variants have appeared. The first variant was D614G, identified in January 2020. The mutation position is close to the S1:S2 processing site that increases the infectivity of the virus (65). Among the variants that emerged subsequently, the World Health Organization (WHO) designated the following as variants of concern (VOC): B.1.1.7 (Alpha), B.1.351 (Beta), P.1 (Gamma), B.1.617.2 (Delta), and B.1.1.529 (Omicron) [Phylogenetic Assignment of Named Global Outbreak lineages (Pangolin) designation] (66). The Alpha variant, containing both N501Y and D614G mutations in the RBD, was discovered in the UK at the end of 2020. N501Y is one of the six ACE2 contact residues. The N501Y mutation increased the affinity of the spike protein for ACE2 (65, 67). The Beta variant, identified in South Africa, has three mutations (E484K, K417N, and N501Y) in the RBD. The mutations of the Brazilian variant Gamma (E484K, K417T, and N501Y) were similar to those of Beta (65). The SARS-CoV-2 B.1.617 lineage was identified in India in October 2020 and includes three main subtypes (B1.617.1, B.1.617.2, and B.1.617.3). B.1.617.2, also termed as the Delta variant, is believed to spread more quickly compared with the other variants and has several key mutations: L452R, P681R, and T478K (66, 68). The Omicron variant, first discovered in Botswana and South Africa in early November 2021, contains more than 30 mutations in the spike protein and is more transmissible than the Delta variant (69). The Omicron variant is subdivided into three sublineages, BA.1, BA.2, and BA.3, of which BA.2 has the strongest transmissibility (70). In addition, other variants have been observed: 20A.EU2 (Spain), COH.20G/677H (Columbus, OH), B.1.427/429 (California), B.1.526 (New York), and B.1.1.298 (Danish mink), etc. (65, 67, 71). The mutations of these variants are mainly in the spike, which are mostly related to increased infectivity and decreased neutralization efficiency, and might escape from humoral immunity acquired *via* prior infections or vaccinations (66).

Although all VOCs are resistant to convalescent serum, the degree of resistance varies among VOC strains. The Omicron variant has the strongest resistance, followed by the Beta, Delta, and Gamma variants, with the Alpha variant having the weakest resistance (44, 66, 72–74). Chen et al. evaluated 106 studies systematically and found that in the live virus neutralization assay, the Beta and Delta variants escaped the neutralization effect mediated by natural infection significantly, showing 4.1-fold and 3.2-fold reductions, respectively, followed by the Gamma and Alpha variants with 1.8-fold and 1.4-fold reductions, respectively (66). One study showed that compared with Victoria (early pandemic virus), the neutralization titers for Omicron variant in convalescent serum from patients previously infected with Alpha, Beta, Gamma, and Delta variants were decreased by 33.8 folds, 11.8 folds, 3.1 folds, and 1.7 folds, respectively (75). A long-term studies have shown consistent results. At 1-year post–SARS-CoV-2 infection, the neutralizing activity of the patient’s serum against Alpha, Beta, B.1.526, and Gamma variants was generally lower than that of against the WT SARS-CoV-2 virus, and the Beta variant had the most loss of activity (33). Compared with the Alpha and D614G strains, the neutralizing titers of the convalescent patients’ serum against Delta at 6 months PSO were significantly reduced by 4 and 6 fold, respectively, which was similar to that of against Beta. At 12 months, the neutralizing activity against Delta and Beta decreased by 4 folds relative to Alpha (68). One study collected convalescent sera at 6 and 12 months PSO, and found that 91% and 94% of them showed neutralizing activity against the Delta variant, whereas only 36% and 39% remained active against the Omicron variant (76).

In addition to the N501Y mutation, the Beta and Gamma variants have two other RBD mutations (E484K and K417N/T), of which E484K affects antibody neutralization resistance to a greater extent. ELISA-based quantification of RBD IgG antibodies showed that in the moderate and severe groups, IgG antibody binding was reduced slightly by the N501Y mutation (1.1 to 1.2 folds), whereas the E484K mutation significantly reduced the binding of IgG antibodies (2.7 to 2.9 folds). Furthermore, K417N and/or N501Y mutations have an additive effect on the E484K mutation. Compared to any RBD mutation alone, the triple mutant RBD caused the most significant reduction (3.7 to 4.0 folds) (44). The L452R substitution in the RBD of the Delta strain might induce structural changes in the binding domain, thereby, promoting the spike-ACE2 receptor interaction. The T478K mutation is unique to the Delta strain and might be related to immune evasion, similar to the E484K mutation in the Beta and Gamma strains. The P681R mutation, located adjacent to the furin cleavage site, has been shown to enhance transmissibility and pathogenicity (68). The Omicron variant has several mutations in RBD, including K417N, T478K, N501Y, E484A shared with Alpha, Beta, Gamma, and Delta variants (70), three mutations close to the furin cleavage site (P681H, H655Y, N679K), six unique mutations in S2, and seven mutations in the NTD (76, 77). The high number of mutations results in high transmissibility and immune evasion ability (77).

The residual neutralizing antibodies, following WT SARS-CoV-2 infection, had less efficacy against variants, particularly, variants possessing strong immune evasiveness, raising concerns regarding re-infection. Studies have confirmed that COVID-19 convalescent patients with detectable neutralizing antibody titers could still be infected by the SARS-CoV-2 variants; thus, they should not be considered safe from the second infection (78).

## Neutralization of vaccine

Since the outbreak of the pandemic, several highly effective vaccines against SARS-CoV-2 have been developed. The widely used vaccines include Live attenuated or inactivated vaccines (BBIBP-CorV and CoronaVac), viral vector vaccines (AZD1222, Ad26COV2-S, and Ad5-nCov), and mRNA vaccines (mRNA1273 and BNT162b2) (79). High seroconversion rates are observed for different vaccines within 14 days of initial vaccination, and nearly all vaccines induced neutralizing antibodies after two doses, with antibody levels similar to or higher than those in convalescent individuals (80–83). Vaccine-induced antibodies naturally wane over time, and the neutralizing activity shows a biphasic decline as in natural infection (80, 84).

Like convalescent sera, many VOCs have reduced susceptibility to vaccine-elicited immunity. The Alpha variant has no apparent effect on vaccine efficacy, but the Beta and Gamma variants could significantly evade vaccine-induced neutralizing antibodies. Serum from individuals who received one dose of vaccine, either the mRNA vaccine (BNT162b2) or the viral vector vaccine (AZD1222), had little inhibitory effect on the Delta variant, but the two doses were still effective, neutralizing 94% and 95% of the Delta variant, respectively (70). Compared to the Delta variant, the neutralizing activity against the Omicron variant was significantly reduced in individuals vaccinated with two doses, and vaccines were significantly less effective against symptomatic COVID-19 infection caused by the Omicron variant (70, 85). Fortunately, current research shows that booster vaccination can reverse the trend of compromised neutralization against Omicron variant (86). In individuals who received three doses of the mRNA vaccines (mRNA-1273 and BNT162b2), only a modest reduction in neutralizing activity was detected compared to the WT virus (87). Furthermore, heterologous booster vaccination appeared to induce higher neutralizing antibody responses than homologous booster vaccination (77). Even without a booster vaccination, most people may be susceptible to mild disease, but still remain long-term protective immunity against severe and fatal disease (70, 88).

Recent research indicated that vaccination of infected patients would induce a higher level of protection (33, 43, 89). Thus, the combination of vaccination and natural infection caused the potency of the antibodies to reach an apparent biological ceiling. Individuals who were vaccinated, approximately 1 year after the initial infection, had relatively unchanged serum neutralization potency toward the original spike sequence; however, the activity against all variants was similar to that of against the original sequence (89). Even against the Omicron variant, a single-dose vaccine can significantly increase the cross-neutralization antibody responses in previously infected individuals (83). These findings support the view that despite COVID-19 convalescent patients retaining a strong specific humoral response against SARS-CoV-2 at 1- year post-infection, further vaccination is still required.

Factors such as age, gender, and comorbidities also affect the level of antibodies elicited by the vaccine. In contrast to natural infection, older individuals, male sex, and those with comorbidities (such as hemodialysis, transplantation, cancer, and autoimmune diseases) showed lower neutralizing antibody levels post-vaccination (90, 91). In addition, individuals with seronegative status had lower antibody titers at each time point after 2 doses of mRNA vaccination compared with the seropositive group (91). However, antibody levels reached peak levels after the second dose in the seronegative group, and a second dose of the vaccine in the seropositive group did not significantly increase antibody titers (91, 92). The rate of decline in humoral responses was consistent 6 months after mRNA vaccination regardless of age, sex, serostatus, and comorbidities (90, 91), suggesting that booster vaccination remains necessary, while the dose and timing of vaccination should be adjusted according to individual characteristics and status.

## Conclusions and future perspectives

The present review summarized the avaialble information regarding the characteristics and dynamics of the specific humoral immune response (IgM, IgA, IgG, and Nabs) elcited by SARS-CoV-2 infection. We also discuss the relationship between the dynamics of neutralizing antibodies and disease severity and the influencing factors. In addition, we briefly summarize the neutralizing potency of infection- and vaccine-elicited humoral immunity against the emerging variants of concern. Such information is critical to design specific diagnostic and therapeutic strategies and should help to optimize public health decisions.

We mainly focus on the changes in antibodies after infection, not cellular immunity, but cellular immunity also plays a key role in various aspects. Antibodies mainly clear extracellular viruses and T cells are required to kill virally infected cells (12). T-cell responses contribute substantially to disease control. Reduced disease severity is related significantly to the timely response of T cells, and patients with persistent COVID-19 show a trend of T cell exhaustion that indicates serious disease progression (93). T cells can also provide long-term immunity. Strong responses of memory T cells to SARS-CoV-2 are detectable months after infection even in the absence of detectable circulating antibodies (94). Unlike antibodies which only RBD-specific antibodies can neutralize, T cells respond to at least 30 epitopes of viral protein. In prior infection, vaccination, both prior infection and vaccinated individuals, and T cell responses are largely preserved to Omicron variants and show robust cross-protection against VOCs (95, 96). Although many VOCs can evade humoral immunity, most vaccines provide protection against severe disease, supporting a major role of cellular immunity in disease control (96).

The importance of cellular immunity is undeniable, but the complexity and cost of measuring cellular immune responses are much higher than routine serological testing of antibodies, which limits the widespread adoption or implementation of cellular immune response assays, and antibody detection remains the primary indicator for assessing immune protection (79, 96). In the future, it is necessary to develop a more convenient cellular immune detection method, quantitatively evaluate antibody levels and cellular immune indicators, calculate the corresponding relationship between immune indicators and immune protection (including prevention of re-infection and critical symptoms), and establish more accurate immune evaluation system.

In infected individuals, neutralizing antibodies decline over time and the protection against breakthrough infection of the variant is diminished, whereas a single dose of vaccination in convalescent individuals can achieve a good effect. In uninfected patients, most vaccines require 2 or even 3 doses to protect against widely prevalent variants. For vulnerable populations, such as the elderly, males, and individuals with comorbidities, the antibody responses caused by natural infection and vaccination are different. For now, there appears to be none of the available vaccines that completely prevent viral infection (79), although the approved SARS-CoV-2 vaccine has a good safety profile, unnecessary vaccination and its side effects should be avoided as much as possible. Further research is needed to assess the extent to which immune protection is weakened over time after infection and vaccination. The use of booster doses and the timing of vaccination should fully consider the individual’s immune efficacy, combined with vaccine coverage and supply, local widespread strains, etc.

## Author contributions

KC and YW conceived the article, YL drafted the article, and JZ generated figures and table. All authors contributed substantially to the content and reviewed or edited the manuscript. All authors approved the manuscript.

## Funding

This work was supported by the opening foundation of the State Key Laboratory for Diagnosis and Treatment of Infectious Diseases, The First Affiliated Hospital, College of Medicine, Zhejiang University, grant NO. SKLID2020KF042.Also, it was supported by the Major horizontal project of Zhejiang Shuren University, grant NO: KHJ1720234.

## Acknowledgments

The authors would like to acknowledge Shulan International Medical College, Zhejiang Shuren University for its support.

## Conflict of interest

The authors declare that the research was conducted in the absence of any commercial or financial relationships that could be construed as a potential conflict of interest.

## Publisher’s note

All claims expressed in this article are solely those of the authors and do not necessarily represent those of their affiliated organizations, or those of the publisher, the editors and the reviewers. Any product that may be evaluated in this article, or claim that may be made by its manufacturer, is not guaranteed or endorsed by the publisher.
