# Supplementary material for: Kinetics of severe acute respiratory syndrome coronavirus 2 infection antibody responses
Source: Front Immunol. 2022 Aug 5;13:864278. doi: 10.3389/fimmu.2022.864278 (PMC9389018; doi:10.3389/fimmu.2022.864278)
Supplement: Supplementary file 1 [file Table_1.docx]

Supplementary Material

# 1. Supplementary Table

| Antibody Class | Detection time | Peak Prevalence | Half-life | | | Duration | | |  |  |
| --- | --- | --- | --- | --- | --- | --- | --- | --- | --- | --- |
| ***IgM*** | | | | | | | | | | |
| *Median* | 7 days | 20 days | 55 - 118 days | | | < 5-6 month | | |  |  |
| *Study, year*  *(reference)* | | Arkhipova-Jenkins I , et al. 2021 (18) | | Wheatley AK , et al. 2021(19) | | Xiang T, et.al. 2021(21); Shi D, et.al. 2021(22) | | | |  |
| ***IgA*** | | | | | | | | | | |
| *Median* | 5-13 days | 16-30 days | 14-210 days | | | 1 year | | |  |  |
| *Study, year*  *(reference)* | Valdez-Cruz NA, et al. 2021(5); Jääskeläinen AJ, et al. 2020(24) Yu HQ, et al. 2020(25) | Isho B, et al. 2020(26); Seow J, et al. 2020(27) | | Dan JM , et al. 2021(28) ; Wheatley AK , et al. 2021(19) | | | | Feng C, et al. 2021(20); Rank A , et al.2021(29); Shi D, et.al. 2021(22) | |  |
| ***IgG*** | | | | | | | | | |  |
| *Median* | 12 days | 25 days | 68-344 days | | | ＞14 month | | |  |  |
| *Study, year*  *(reference)* | Arkhipova-Jenkins I , et al. 2021 (18) | | | Dan JM , et al. 2021(28) ; Terpos E , et al. 2021(31); Grandjean L, et al. 2021(32); Wheatley AK , et al. 2021(19) | | | Li C, et al. 2021(15); Wang Z, et al. 2021(33); Choe PG, et al. 2021(34); Choe PG, et al. 2021(35); Rank A , et al. 2021.(29); Guzmán-Martínez O (36); Feng C, et al.(20); Gallais F, et al. 2021(37); Xiang T, et.al. 2021(21); Shi D, et.al. 2021(22); Dehgani-Mobaraki P, et al. 2021 (38) | | |  |
| ***Neutralizing antibodies*** | | | | | | | | | |  |
| *Median* | 6–15 days | 14–45 days | 27-519 days | | | ＞13 months | | |  |  |
| *Study, year*  *(reference)* | Chao YX, et al. 2020 (22) | Isho B, et al. 2020(26) | | Dan JM , et al. 2021 (28) ; Chao YX, et al. 2020 (23); Choe PG, et al. 2021 (18); Wheatley AK , et al. 2021(19); Terpos E , et al. 2021 (31) | | | Xiang T, et.al. 2021(21); Rank A, et al.2021(29); Choe PG, et al. 2021 (18); Haveri A, et al. 2021 (42) Shi D, et.al. 2021(22) | | |  |

**Supplementary Table 1** Kinetics of SARS-CoV-2 Infection antibody responses
